# Megalin/LRP2 Expression Is Induced by Peroxisome Proliferator-Activated Receptor -Alpha and -Gamma: Implications for PPARs' Roles in Renal Function

**DOI:** 10.1371/journal.pone.0016794

**Published:** 2011-02-02

**Authors:** Felipe Cabezas, Jonathan Lagos, Carlos Céspedes, Carlos P. Vio, Miguel Bronfman, María-Paz Marzolo

**Affiliations:** 1 Departamento de Biología Celular y Molecular, Facultad de Ciencias Biológicas, Pontificia Universidad Católica de Chile, Santiago, Chile; 2 Millenium Nucleus in Regenerative Biology (MINREB), Pontificia Universidad Católica de Chile, Santiago, Chile; 3 Departamento de Fisiología, Pontificia Universidad Católica de Chile, Santiago, Chile; Chinese University of Hong Kong, Hong Kong

## Abstract

**Background:**

Megalin is a large endocytic receptor with relevant functions during development and adult life. It is expressed at the apical surface of several epithelial cell types, including proximal tubule cells (PTCs) in the kidney, where it internalizes apolipoproteins, vitamins and hormones with their corresponding carrier proteins and signaling molecules. Despite the important physiological roles of megalin little is known about the regulation of its expression. By analyzing the human megalin promoter, we found three response elements for the peroxisomal proliferator-activated receptor (PPAR). The objective of this study was to test whether megalin expression is regulated by the PPARs.

**Methodology/Principal Findings:**

Treatment of epithelial cell lines with PPARα or PPARγ ligands increased megalin mRNA and protein expression. The stimulation of megalin mRNA expression was blocked by the addition of specific PPARα or PPARγ antagonists. Furthermore, PPAR bound to three PPAR response elements located in the megalin promoter, as shown by EMSA, and PPARα and its agonist activated a luciferase construct containing a portion of the megalin promoter and the first response element. Accordingly, the activation of PPARα and PPARγ enhanced megalin expression in mouse kidney. As previously observed, high concentrations of bovine serum albumin (BSA) decreased megalin in PTCs *in vitro*; however, PTCs pretreated with PPARα and PPARγ agonists avoided this BSA-mediated reduction of megalin expression. Finally, we found that megalin expression was significantly inhibited in the PTCs of rats that were injected with BSA to induce tubulointerstitial damage and proteinuria. Treatment of these rats with PPARγ agonists counteracted the reduction in megalin expression and the proteinuria induced by BSA.

**Conclusions:**

PPARα/γ and their agonists positively control megalin expression. This regulation could have an important impact on several megalin-mediated physiological processes and on pathophysiologies such as chronic kidney disease associated with diabetes and hypertension, in which megalin expression is impaired.

## Introduction

Megalin/LRP2 is a large membrane glycoprotein that belongs to the low-density lipoprotein receptor (LDLR) family, which includes LDLR, LRP8 (apolipoprotein E receptor 2) very low-density lipoprotein receptor, LDLR-related protein 1 (LRP1) and LRP1B [1]. This multiligand endocytic receptor is expressed on the apical membrane of several epithelial cells, including those of the kidney proximal tubule cells (PTCs), lung, thyroid [2], gallbladder [3] and neuroepithelium [4], [5]. The ectodomain of megalin/LRP2 is very large and is responsible for ligand binding. In contrast, the cytoplasmic domain of the receptor is relatively small and contains three NPXY motifs, which have been suggested to regulate its endocytic activities [6]. In addition, the megalin cytoplasmic domain determines the apical localization of the receptor [7] and can be phosphorylated by glycogen synthase kinase 3(GSK3), an event that regulates megalin recycling and cell surface availability [8].

Megalin's functions cover a broad spectrum and include processes that are critical during development [4], [5], [9], [10], [11] and adult life and are impaired in several pathologic conditions that compromise the kidney and the central nervous system [12], [13], [14]. In addition, it has been recently suggested that megalin could have roles in regeneration processes in both peripheral and central nervous systems as well as in kidney [15], [16], [17], [18], [19], [20]. One of the best studied systems of megalin function is the proximal tubule, where the receptor is able to recover and internalize several molecules, including complexes of vitamin A, B12 and D, along with their corresponding transporter proteins [21], leptin [22], angiotensin II (and I-VII) [23], [24], insulin [25] and albumin [12].

Megalin expression is compromised in kidney disease associated with diabetes [26], [27] and other conditions, such as aging [28]. However, the molecular mechanisms underlying downregulation of this receptor remain obscure. Additionally, little is known about the transcriptional mechanisms regulating the physiological expression of the megalin gene. Previous studies have indicated a positive regulatory role of cyclic AMP, retinoic acid and vitamins A and D in the expression of megalin in cell culture [29], [30]. Angiotensin II and insulin have also been reported to be involved in the regulation of megalin expression in opossum proximal tubule cells (OK) [31]. Finally, although the mechanism is unclear, high concentrations of albumin significantly reduced the expression of megalin in PTCs *in vitro* [32].

Peroxisome proliferator-activated receptors (PPARs) are transcription factors belonging to the nuclear receptor superfamily, for which three isoforms (α, β/δ and γ) that are encoded by separate genes have been described [33]. PPARs form active heterodimers with the 9-cis-retinoid X receptor (RXR) and bind to characteristic DNA sequences, the PPAR-responsive elements (PPREs) that are located in the promoters of their target genes [34]. Fatty acids and their oxidized metabolites have been proposed to be natural PPAR ligands [35], [36], [37]. Moreover, synthetic ligands for PPARα and PPARγ such as fibric acid and thiazolidinediones, have positive effects in human diseases, including type 2 diabetes, metabolic syndrome, obesity and insulin resistance [38]. It is also known that PPARs regulate the expression of some LDLR family receptors, with LRP1 expression induced by PPARγ [39] and VLDL-R expression regulated by PPARα [40].

Megalin is highly expressed in a pattern similar to the three PPAR subtypes in the kidney [41], [42] and in PTC lines [43]. In addition, both PPARα and PPARγ agonists have renoprotective actions that are independent of their systemic roles related to metabolic control [38]. A long-term study of human patients with type 2 diabetes showed that treatment with the PPARα agonist fenofibrate significantly reduced albuminuria [44], which could be potentially related to megalin function in the kidney, including its function as an albumin receptor. In addition, the PPARα agonists gemfibrozil and fenofibrate were able to reduce kidney hypertrophy and fibrosis associated with diabetic nephropathy in two different diabetic animal models [45], [46]. Furthermore, the beneficial effect of gemfibrozil on the kidney was shown to be independent of the lipid-lowering effect of the drug [45]. The dual PPARα/γ agonists, JTT-501 [47] and compound 3q [45], are also renoprotective agents in both mouse and rat diabetic models. Moreover, in diabetic patients and diabetic animal models, which exhibit reduced megalin expression [26], [27], it has been shown that several PPARγ agonists decreased albuminuria [45], [47], [48], [49], [50], [51], [52]. It has also been reported that the treatment with the PPARγ agonist pioglitazone increased tubular cell albumin uptake *in vitro* [43]. However, in these studies, the mechanisms by which the synthetic PPAR agonists reduced the albuminuria and promoted albumin uptake in PTCs were not completely clear and likely involved glomerular as well as tubular effects.

In this study, we tested the hypothesis that megalin expression is regulated by PPARs. We found three consensus PPREs in the human megalin promoter. In addition, we show evidence that PPARα, PPARγ and their corresponding ligands induce megalin expression at the mRNA and/or protein level in cell lines and in the kidney *in vivo*. Using *in vitro* PTC models of BSA-induced downregulation of megalin expression, we demonstrate that treatment with either PPARα or PPARγ agonist ameliorates the reduction in megalin expression. This protective effect was also observed when PPARγ agonists were used *in vivo*. Overall, our findings suggest that PPARs and their agonists could be critical regulators of megalin expression in various pathophysiological conditions, including chronic renal disease.

## Results

### The presence of PPREs in the megalin promoter and the participation of PPARα and PPARγ, but not PPARβ/δagonists in megalin expression

**Figure 1:**
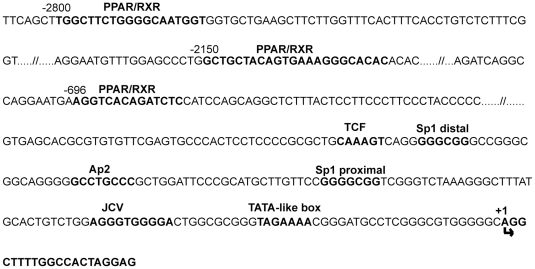
Putative PPAR consensus sites in the human megalin promoter. Nucleotide numbering is labeled relative to the transcription start site (nt +1, arrow). Putative PPAR responsive-elements (PPREs) were located by MatInspector (www.genomatix.de).

The analysis of the human megalin promoter sequence (NC_000002.11) using MatInspector revealed the presence of three putative PPREs, located at −696 bp (AGGTCACAGATCTC), −2150 bp (GCTGCTACAGTGAAAGGGCACAC) and −2800 bp (TGGCTTCTGGGGCAATGGT) relative to the transcriptional start site (Fig. 1). This finding prompted us to determine whether megalin expression responds to distinct PPAR agonists in *in vivo* and *in vitro* models.

**Figure 2:**
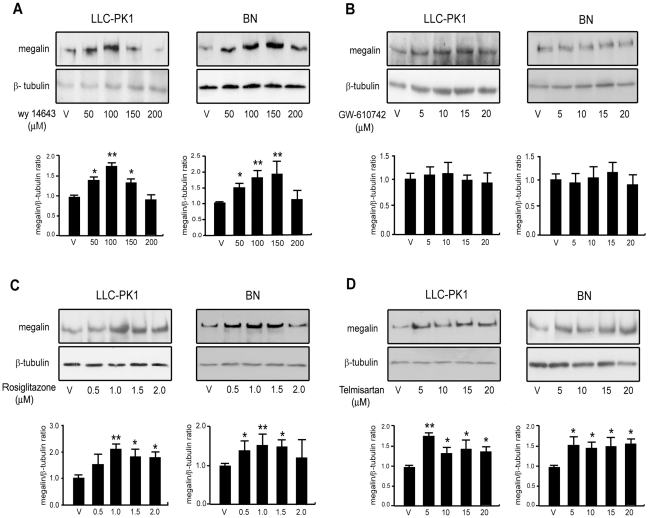
Megalin protein levels are increased in BN and LLC-PK1 cultured cells upon exposure to PPARα and PPARγ agonists, but not PPARβ agonists. (**a**) BN cells (600,000 cells/well) and LLC-PK1 cells (500,000 cells/well) were treated with different concentrations of the PPARα agonist WY 14643, (**b**) the PPARβ agonist GW 610742, and the PPARγ agonists (**c**) rosiglitazone and (**d**) telmisartan for 24 h in 0.5% FBS in DMEM. Cells were lysed and the expression of megalin and the loading control, β-tubulin, was determined by western blot. The bands in the blots were quantified by densitometry, and the results were plotted as the ratio of megalin/β-tubulin for each condition. Statistically significant differences compared to the control (DMSO) are indicated as *P<0.05, **P<0.01.

First, we determined baseline megalin expression levels in LLC-PK1 cells, an epithelial cell line derived from kidney proximal tubule, and in BN cells, a cell line derived from rat yolk sac. The expression of the three PPAR subtypes has previously been documented in kidney [41], [42] and proximal tubule cell lines, including LLC-PK1 cells [53], [54], [55]. Additionally, we confirmed the expression of PPARα, β and γ in BN cells by RT-PCR (Fig. S1). Next, cells were treated with varying concentrations of either WY 14643 (a potent PPARα agonist), GW 610742 (a PPARβ/δ agonist), telmisartan (an angiotensin II type-1 receptor (AT1) blocker that is also a PPARγ modulator [56], [57]) and rosiglitazone (a synthetic agonist for PPARγ). After treatment for 24 h, cells were lysed, and megalin and β-tubulin protein levels were determined by western blot. Treatment with WY 14643 led to a dose-dependent increase in the expression of megalin, which was significant starting at concentrations of 50 µM in both cell lines, and a reduction in expression towards control levels at higher doses of 200 µM (Fig. 2A). In contrast to the effect of the PPARα agonist WY 14643, agonists for PPARβ/δ did not affect megalin expression in either cell line (Fig. 2B). The effect of PPARγ agonists was evaluated using rosiglitazone (Fig. 2C) and telmisartan (Fig. 2D). Both agonists were able to significantly induce the expression of megalin in BN and in LLC-PK1 cells.

### Endogenous megalin mRNA expression responses to PPARα and PPARγ agonists and antagonists

**Figure 3:**
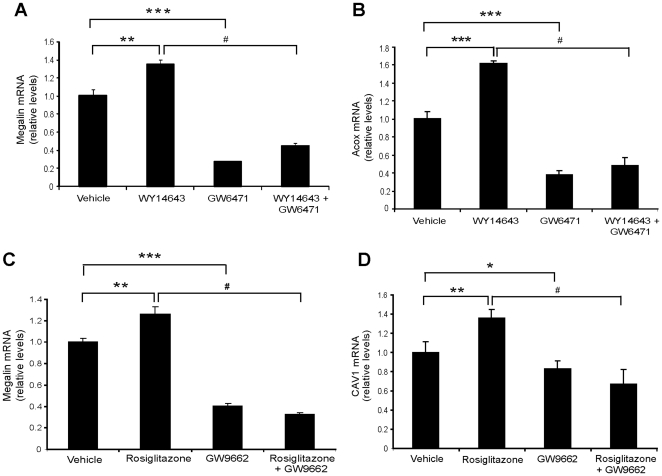
PPARα and PPARγ agonists or antagonists regulate megalin mRNA expression in LLC-PK1 cells. (**a, b**) LLC-PK1 cells were treated with 100 µM WY 14643 (PPARα agonist), 10 µM GW 6471 (PPARα antagonist) or both drugs for 24 h. Megalin and Acox mRNA levels were determined by qPCR, and values were normalized to the housekeeping gene actin (n = 4). (**c, d**) LLC-PK1 cells were exposed to 1 µM rosiglitazone (PPARγ agonist), 10 µM GW 9662 (PPARγ antagonist) or both drugs for 24 h. Megalin and caveolin 1 mRNA levels were detected by qPCR, and values were normalized to the signal from the housekeeping gene actin (n = 4). Results are expressed as means ± standard deviation (SD). Statistically significant differences compared to the control (DMSO, vehicle) are indicated by *P<0.02, **P<0.01, ***P<0.001. Statistically significant differences to the agonist alone are indicated by ^#^P<0.001.

We determined if PPARα, PPARγ and their ligands could induce megalin expression at a transcriptional level by qRT-PCR. When LLC-PK1 cells were treated with agonists (100 µM WY 14643 or 1 µM rosiglitazone) for 24 h, megalin mRNA levels were significantly induced. In contrast, when cells were only incubated with an antagonist for either PPARα (GW 6471) or PPARγ (GW 9662), the expression of megalin was significantly reduced compared to the control condition, indicating that an important aspect of the regulation of basal megalin transcription may be mediated by PPARα/γ ligands, such as fatty acids, present in the serum. Interestingly, when cells were pre-incubated with the PPARα and PPARγ antagonists, the effects of the agonists WY 14643 and rosiglitazone were inhibited (Fig. 3A, C). As known PPAR target genes, we determined the expression of Acox and caveolin 1 after stimulation of PPARα and PPARγ, respectively (Fig. 3B, D).The expression of both genes was altered as expected after treatment with the appropriate agonists/antagonists. These results suggest that both PPARα and PPARγ nuclear receptors are involved in the activation of megalin gene transcription.

### Megalin expression is increased *in vivo* by treatment with PPARα and PPARγ activators

**Figure 4:**
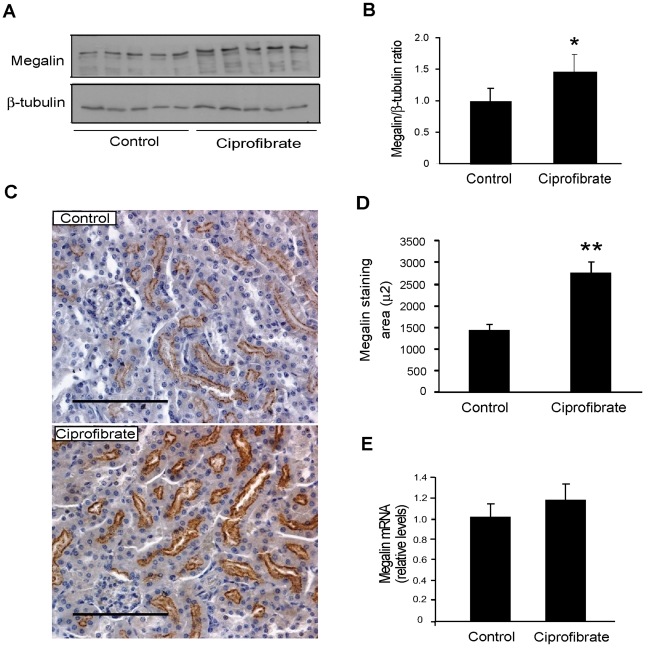
PPARα agonists regulate megalin expression *in vivo* in mice. BALB/c mice (n = 3–5/per group) received ciprofibrate (200 mg/kg/day) or vehicle for 1 week. (**a**) Kidney extracts were used to determine megalin protein levels by western blot, with β-tubulin used as loading control. (**b**) The bands in the blots were quantified by densitometry, and the results were plotted as the ratio of megalin/β-tubulin for each condition. (**c**) Immunohistochemical staining for the megalin cytoplasmic domain in kidney sections (cortex) showed increased staining for megalin in ciprofibrate–treated mice compared to control mice. Bar = 100 µm. (**d**) The megalin-immunostained area was significantly increased in the ciprofibrate group compared to the control group as measured by morphometric analysis. The quantification of the megalin-stained area is shown as µm^2^. (**e**) Megalin mRNA expression was determined from kidney RNA by qPCR. Data are expressed as means ± SD. *P<0.05 vs. control, **P<0.0001 vs. control.

**Figure 5:**
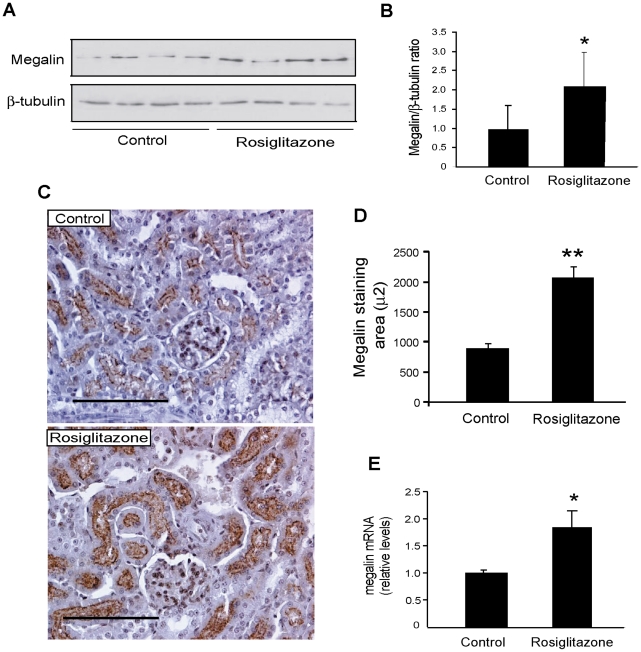
PPARγ agonists regulate megalin expression *in vivo* in mice. BALB/c mice (n = 4–6/per group) received rosiglitazone (20 mg/kg/day) or vehicle for 10 days. (**a**) Kidney extracts were used to determine megalin protein levels. β-tubulin was used as loading control. (**b**) The bands in the blots were quantified by densitometry, and the results were plotted as the ratio of megalin/β-tubulin for each condition. (**c**) Immunohistochemical staining of megalin in kidney sections (cortex) showing increased immunostaining for megalin in rosiglitazone-treated mice compared to control mice. Renal tissue was immunostained using an antibody that detects the megalin cytoplasmic domain. Bar = 100 µm. (**d**) The megalin-immunostained area was significantly increased in the rosiglitazone group compared to the control group as measured by morphometric analysis. The graph shows the quantification of the megalin-stained area in µm^2^. (**e**) Megalin mRNA levels were determined by qPCR. Data are expressed as means ± SD. *P<0.05 vs. control, **P<0.0001 vs. control.

Because PPARs agonists induced the expression of megalin in cell culture, we evaluated whether these compounds were also able to increase megalin expression *in vivo* in mice. BALB/c mice were treated with the PPARα ligands ciprofibrate and WY 14643 or the PPARγ ligands rosiglitazone and telmisartan (for details, see Table S3). Activation of PPARα with ciprofibrate significantly induced the expression of megalin in the mouse kidney at the protein level, as measured both by western blot (Fig. 4A, B) and by immunohistochemistry (Fig. 4C, D). In contrast, this treatment did not modify the expression of megalin at the mRNA level (Fig. 4E) although the PPARα activator treatment did effectively alter expression of the target gene Acox (Fig. S2A). Treatment with WY 14643 also upregulated the expression of Acox mRNA (Fig. S2A), but similar to ciprofibrate treatment, was unable to induce megalin expression at the mRNA level (Fig. S2B). However, the expression of megalin protein, as determined by western blot, was significantly increased by WY 14643 (Fig. S2C, D). In contrast, treatment with the PPARγ activator rosiglitazone significantly increased the expression of megalin at both the protein and mRNA levels (Fig. 5A–E). Similar to rosiglitazone, treatment with Telmisartan, a partial PPARγ agonist, resulted in the induction of megalin at protein level in mice (Fig. S3A, B) and in the kidney of rats, as determined by both IHQ (Fig. S3C, D) and qPCR (Fig. 9A).

### Activation of a megalin promoter construct containing the putative PPRE, located at -696 bp relative to the transcription initiation, by PPARα and its agonist in a Luciferase/β-galactosidase Reporter Assay

**Figure 6:**
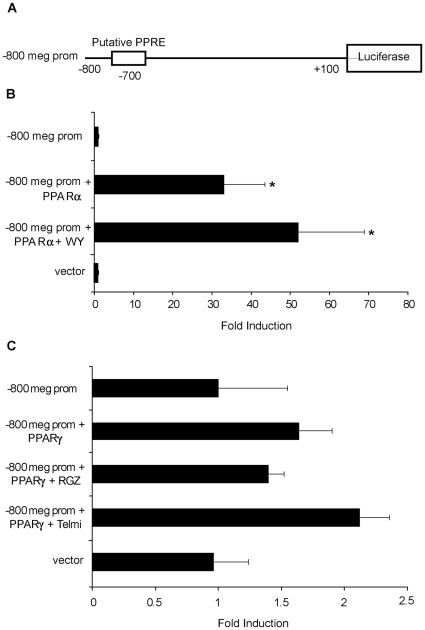
The first PPRE of the megalin promoter is activated by PPARα, but not PPARγ. (**a**) Schematic of the construct used to analyze megalin promoter activity. (**b**) BN cells (6×10^4^ cells) were seeded in 24-well plates in triplicate. The cells were transfected 24 h later with the megalin promoter construct (−800 meg-Luc) and a β-galactosidase vector (pCMVβ). Cotransfections were performed by adding either PPARα or PPARγ cDNAs. After 24 h, the transfected cells were treated with specific agonists for the PPARs and incubated for an additional 24 h prior to assaying the luciferase activity. Cells expressing the megalin promoter construct were able to increase luciferase activity 30-fold when PPARα was overexpressed and nearly 50-fold when in addition to PPARα, cells were treated with 100 µM WY 14643. (**c**) In contrast, overexpression of PPARγ with or without the corresponding ligands and treatment with rosiglitazone or telmisartan was unable to induce luciferase expression. Data are expressed as means ± SD. *P<0.05 vs. the −800 meg-Luc control.

We next investigated whether a luciferase reporter gene construct containing an 800-bp region of the human megalin promoter sequence and the first PPRE was able to respond to PPAR transcription factors and their ligands (Fig. 6A). BN cells expressing the megalin promoter construct were able to induce luciferase expression by 30-fold when coexpressing exogenous PPARα and by nearly 50-fold when they were also incubated with the PPARα agonist, WY 14643 (Fig. 6B). In contrast, overexpression of PPARγ with or without its ligands (rosiglitazone or telmisartan) was unable to induce luciferase expression (Fig. 6C). Similar results were obtained when LLC-PK1 cells were used (data not shown). These results suggest that the first PPRE of the human megalin promoter is targeted by PPARα, but not PPARγ.

### Binding of PPARα-RXRα and PPARγ-RXRα heterodimers to PPREs identified in the megalin promoter region

**Figure 7:**
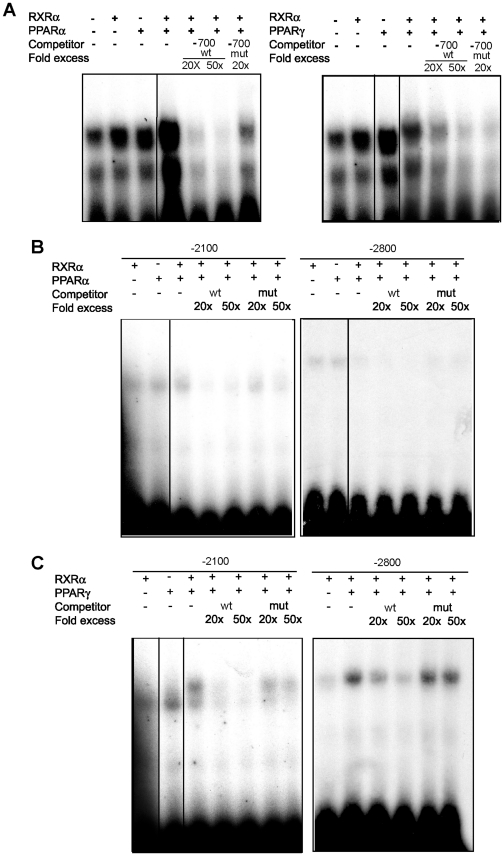
Binding of PPAR/RXRα heterodimers to the PPREs in the human megalin promoter. EMSAs were performed with *in vitro* translated RXRα plus PPARα or PPARγ using probes for the wild-type (wt) PPREs of the human megalin promoter. The competition analyses were performed with a 20- or 50-fold excess of the wt or mutated probes. (**a**) −700 bp, (**b**) −2150 bp and (c) −2800 bp. PPARα bound to all the sites, whereas PPARγ bound specifically to the −2150 and −2800 bp sites.

We tested the binding ability of the three PPRE consensus sites in the human megalin promoter using electrophoretic mobility shift assay (EMSA) reactions (Fig. 1). For the first PPRE, located at −696 to −683, we observed specific binding of a PPARα-RXRα heterodimer. In contrast, we did not find any specific binding for PPARγ because an excess of either the wild-type or the mutant unlabeled oligomers were able to displace the ^32^P-end-labeled DNA from its binding to the heterodimer (Fig. 7A). These results are in agreement with the promoter activation assays, which indicated that PPARα, but not γ, was able to activate the PPRE present in this promoter region. PPARγ agonists activated megalin expression both *in vivo* and *in vitro*; thus, we hypothesized that the other two PPREs at positions – 2800 and -2150 might be able to bind the PPAR-RXRα protein complexes. In accordance with this hypothesis, both PPARα and PPARγ specifically bound to the labeled oligomer that corresponded to the -2150 promoter region (Fig. 7B, C). This binding could be competed away by a 20- to 50-fold increase of the wild-type, but not the mutant, PPRE oligomer. PPARα binding to the -2800 PPRE region was somewhat weak in our experimental conditions but still could be displaced by an excess of the appropriate unlabeled oligomer. However, the binding of PPARγ to this region was clear and specific. We did not detect binding of PPARβ/δ-RXRα heterodimers to any of the sequences analyzed (data not shown), which is in agreement with our data demonstrating that treatment with PPARβ agonist did not increase megalin expression (Fig. 2). Overall, these results indicate that PPARα and PPARγ bind to multiple PPREs in the megalin promoter, which supports our observations of activated megalin expression in the various model systems treated with PPAR agonists.

### Megalin expression is reduced by high concentration of albumin: the role of PPARα and PPARγ agonist treatments

**Figure 8:**
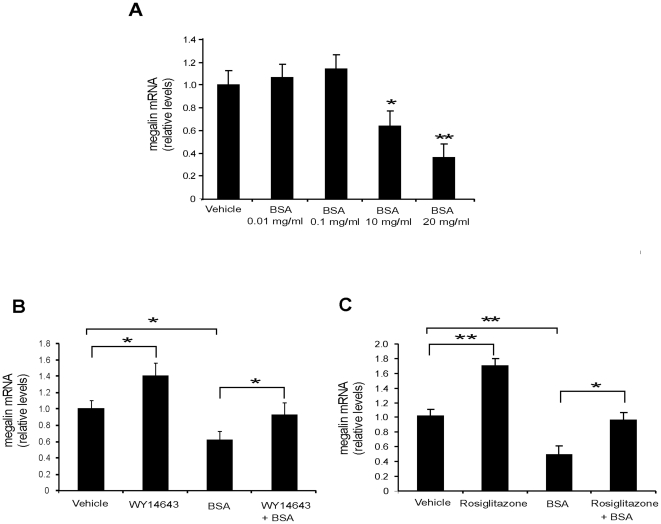
PPARα and PPARγ agonists have a protective role against BSA-induced megalin downregulation in cultured PTCs. LLC-PK1 cells were seeded and used 2 days after confluence was reached. Before use in experiments, cells were cultured in medium depleted of serum for 16 h. Each condition was assayed in triplicate. (**a**) Cells were incubated with different concentrations of BSA for 24 h. Megalin mRNA expression was determined by qPCR. At concentrations of 10 mg/ml, BSA induced a significant reduction in megalin mRNA expression (n = 3). (**b, c**) LLC-PK1 cells were pretreated with 100 µM WY 14643 (PPARα agonist), 1 µM rosiglitazone (PPARγ agonist) or vehicle for 1 h prior to 20 mg/mL BSA exposure, which was maintained for 24 h. Megalin mRNA levels were determined from cellular RNA extracts by qPCR. Values were normalized to the expression of the housekeeping gene of actin (n = 3–4). Results are expressed as means ± standard deviation (SD). Statistically significant differences are indicated as *P<0.05, **P<0.01.

Reduced megalin immunoreactivity has been previously described in diabetic rats with albuminuria [26]. In addition, high doses of BSA were associated with a decrease in megalin expression in PTCs *in vitro*, but the mechanism for this observation was not determined [32]. Based on these studies, we reasoned that the treatment of LLC-PK1 cells with high concentrations of BSA combined with the effect of PPARα and PPARγ agonists on megalin expression would be a good model to initially test for a protective role of these nuclear receptors on megalin expression. We found that megalin expression was significantly reduced in cells that were exposed to BSA concentrations over 10 mg/ml (Fig. 8A), similar to previously published data [32]. When cells were exposed to agonists for PPARα (WY 14643) or PPARγ (rosiglitazone), megalin mRNA levels significantly increased (Fig. 3, Fig. 8B–C). Interestingly, when the cells were treated with PPAR agonists and BSA (10 mg/ml) simultaneously, both agonists were able to significantly counteract the inhibitory effect of BSA on megalin expression.

**Figure 9:**
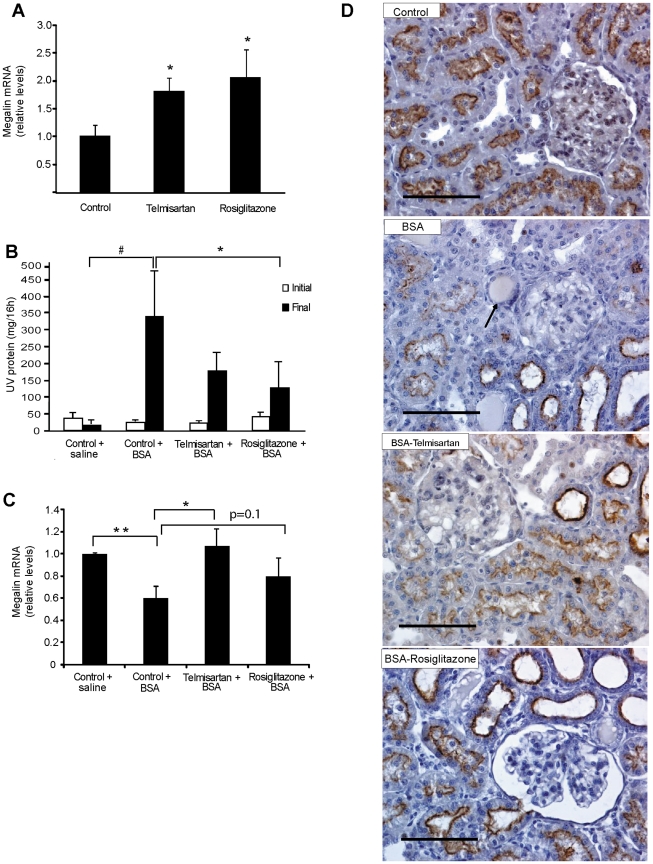
PPARγ agonists have a protective role in BSA-induced tubulointerstitial damage, decrease proteinuria and prevent downregulation of megalin. (**a**) The expression of megalin mRNA, determined by qPCR, in the kidneys of rats treated with telmisartan or rosiglitazone for 4 days was increased significantly compared to the control (vehicle); *P<0.05 vs. control. (n = 4 per group). (**b**) A separate group of rats continued with the treatments for one week and simultaneously received daily injections of 2 g BSA or saline. Before (Initial) and after (Final) the BSA injections, proteinuria (UV prot) was determined as explained in the Material and Methods. Pretreatment with agonists alone did not modify the UV prot levels (comparison of all groups at Initial conditions); however, rosiglitazone decreased the proteinuria induced by the BSA injections. ^#^P<0.001 vs. final control + saline. *P<0.05 vs. final control + BSA. (**c**) At the end of the treatments, the expression of megalin was determined by qPCR. BSA induced a significant downregulation of megalin mRNA compared to the control and both PPARγ agonists maintained megalin mRNA levels close to the control condition without BSA. **P<0.01 vs. control + saline, *P<0.05 vs. control + BSA. (**d**) Immunohistochemical staining for megalin in kidney sections (cortex). In control animals, megalin protein is abundantly expressed in the apical pole of proximal tubule cells (PTCs), whereas the BSA-treated animals exhibited a decrease in megalin immunostaining of the apical pole of PTCs, especially in areas where tubular dilatation with intraluminal proteinaceous casts were evident (arrow). Kidneys from animals treated with BSA and PPARγ agonists show a partial recovery of megalin immunostaining along with the presence of less tubular damage. Representative figures from the different groups are shown. Bar = 100 µm.

We next moved to an *in vivo* model; we induced proteinuria in healthy rats by daily administration of 2 g BSA for one week, which resulted in downregulation of megalin expression, similar to the *in vitro* observations. To test whether activation of PPARγ was able to prevent the reduction of megalin in kidney, three groups of four animals received vehicle, telmisartan or rosiglitazone for four days. During this “pretreatment” condition, the expression of megalin was determined by qPCR and found to be significantly increased in the kidneys of the animals receiving telmisartan or rosiglitazone compared to the controls (Fig. 9A). In parallel, four other groups of animals, including two controls pretreated with vehicle, started receiving daily injections of BSA (or saline as control) for 7 days to induce tubulointerstitial damage as previously described [58], [59], [60]. Before and after the BSA administration, renal function parameters were determined. Creatinine clearance was similar in all experimental conditions (Table 1), but rats in the BSA-treated group developed significant proteinuria (Fig. 9B, Table 1) that was significantly lower in the animals treated with rosiglitazone, indicating a protective role for PPARγ in this experimental condition (Fig. 9B). Megalin mRNA levels were also significantly lower in the BSA-treated animals receiving vehicle (Fig. 9C); however, in the rats receiving telmisartan the negative effect of BSA on megalin expression was avoided, and in the animals receiving rosiglitazone the effect of BSA was partially neutralized. In both cases megalin mRNA expression were maintained at levels similar to the control animals (saline) (P = 0.81, control + saline vs. Telmisartan + BSA; P = 0.38, control + saline vs. rosiglitazone +BSA). Moreover, in the Telmisartan + BSA group, megalin mRNA was significantly upregulated compared the control + BSA group; a similar, but not significant, tendency was observed in the rosiglitazone + BSA group (P = 0.1, vs. control + BSA). The effects of the BSA injections and PPARγ agonist treatments were also established by morphological examination. The BSA-treated animals exhibited marked tubular lesions within the proximal and distal tubules, including epithelial flattening, tubular dilatation, intraluminal proteinaceous casts and interstitial infiltration of mononuclear cells, without evidence of fibrosis. In addition, a clear reduction in immunoreactive renal megalin located at the luminal pole of proximal cells was evident in BSA-treated animals compared to control animals, especially in the tubules showing intraluminal proteinaceous casts (Fig. 9D). Consistent with the alterations in mRNA levels, megalin immunoreactivity was less decreased in the BSA-treated animals receiving PPARγ agonists compared to the BSA-treated animals receiving vehicle (Fig. 9D). Overall, these results indicate that the reduction in megalin expression observed in kidneys exposed to high doses of BSA is partially, but significantly, prevented by treatment with PPARγ agonists.

**Table 1:** Physiological data of BSA injected rats.

		Vehicle+ saline	Vehicle + BSA	Telmisartan + BSA	Rosiglitazone + BSA
Body weight (g)	Initial	193.0±12	189.7±10	183.3±5.0	187.0±6.1
	Final	225.3±18	234.7±7.4	213.7±25	230.7±14
Creatinine clearance (ml/min)	Initial	0.85±0.12	0.93±0.04	1.04±0.18	1.16±0.07
	Final	1.21±0.31	0.96±0.16	1.16±0.24	1.23±0.20
Proteinuria (mg protein/mg creatinine)	Initial	9.75±2.57	5.96±2.03	5.63±0.87	9.16±0.99
	Final	4.24±3.95	73.27±24.4 #	37.22±7.7	25.58±11.9 *
Serum creatinine (mg/dl)	Initial	0.47±0.06	0.47±0.06	0.40±0.00	0.40±0.00
	Final	0.43±0.06	0.50±0.00	0.43±0.06	0.40±0.00

Sprague-Dawley rats, were pretreated with Telmisartan (3 mg/k/day, n = 4), Rosiglitazone (3 mg/k/day; n = 4) or vehicle (in this case n = 8) for 4 days. From day 5 to 11 the animals continued with the treatment and were, in addition, injected with sterile saline or BSA (2 g/Day) as indicated. Creatinine clearance, serum creatinine and proteinuria were determined as described in Methods on the day 5 and 11 of the experiment.

**#**P<0.001 (vs Control + Saline);

*P<0.05 (vs Control + BSA).

## Discussion

The fundamental role of megalin in kidney and in development is clearly demonstrated by the phenotype of megalin knockout mouse models [4], [5], [61], [62]. The absence of megalin expression in the kidney is associated with the urinary loss of ligands, including albumin and low molecular weight proteins such as vitamin D-binding protein and retinol-binding protein. Kidneys of megalin knockout animals showed a significant reduction in the endocytic compartments of PTCs, including clathrin-coated pits and apical recycling compartments [61]. In addition, it has recently been suggested that megalin expression is decreased in the kidneys of old rats [28], which could potentially affect the renal functions in which megalin is involved. Moreover, there are pathological conditions, such as Dent's Disease and Lowe Syndrome, in which megalin expression is functionally compromised [14], [63], [64]. However, despite the important roles of the megalin receptor, there are few studies that have investigated the mechanisms that control its expression.

In this study, we have shown for the first time, using both *in vitro* and *in vivo* models, that megalin expression is induced by PPARα and PPARγ agonists at the mRNA and/or protein levels. We found that specific antagonists for PPARα and PPARγ disrupted the activation effect of WY 14643 and rosiglitazone, respectively, which strongly suggests a direct role of PPARα/γ in the regulation of megalin expression. In addition, several lines of evidence support the role of both nuclear receptors in the activation of megalin expression. First, overexpression of PPARα activates expression of a luciferase gene under the control of a portion of the human megalin promoter containing one PPRE. Second, the effect of telmisartan, which binds directly to PPARγ [65]. Moreover, we showed that PPARα and PPARγ heterodimers containing RXR bind to at least two of the three PPREs present in the human promoter of megalin, which is a potential mechanism for the activation of megalin expression observed at the transcriptional level in our animal and cellular models.

We tested the hypothesis that megalin is a novel PPARα and PPARγ target gene in order to determine whether these nuclear receptors and their agonists could have a protective role in conditions in which there is reduced megalin expression using *in vitro* and *in vivo* methodologies. Our strategy was based on the data previously published by Caruso-Neves *et al.* [32], which showed that LLC-PK1 cells incubated with high concentrations of BSA have a significant reduction in megalin at both the mRNA and protein levels. Although the mechanisms involved in the BSA-mediated reduction of megalin were not completely clear, this study proposed that megalin normally has a protective role in PTCs based on its direct association to PKB/AKT in conditions that are associated with high concentrations of albumin and because the severely reduced megalin expression levels were correlated with the induction of PTC apoptosis [32]. A similar situation could occur *in vivo* if the barrier function of the glomerulus was disrupted, resulting in excess filtration of albumin and induction of renal damage. Interestingly, we found that the pretreatment of LLC-PK1 cells with either PPARα or PPARγ agonists prevented the drastic reduction of megalin using similar methods to the described *in vitro* experimental conditions [32]. We next tested whether megalin expression in PTCs *in vivo* could also be downregulated by an excess of filtered albumin and whether expression could be recovered by PPARγ agonists. Our results demonstrated that treatment with PPARγ agonists circumvented the significant reduction of megalin in PTCs from BSA-injected rats and also reduced the proteinuria that had developed in these animals. These observations may provide a mechanistic context to the known protective roles of PPARα/γ in kidney diseases, such as diabetic nephropathy, which is associated with proteinuria and compromised expression of megalin. In addition, recent studies indicate that megalin expression is reduced in conditions associated with proteinuria. For example, megalin expression was reduced in the Ren2 rat, a model used for studying hypertension and metabolic syndrome [66]. Treatment of Ren2 rats with a β1-antagonist blocker to reduce the secretion of renin improved the reabsorption of albumin and LMW proteins by the PTCs and restored megalin expression levels. Our data suggest that treatment with the PPARγ partial agonist/AT1 antagonist telmisartan would also have a beneficial effect in this model, inducing megalin expression that could otherwise be decreased by angiotensin II with hypertensive conditions [31].

Similar to PPARγ agonists, treatment with PPARα agonists has been linked to a reduction in proteinuria [46] and to the regulation of the renin-angiotensin system, with a reduction of AT1 and the induction of AT2 expression in the kidney [67]. Furthermore, it has been shown that renal disease, including albuminuria, is more severe in diabetic mice that are genetically deficient for PPARα than in wild-type mice [68]. Although we did not test PPARα agonists in our protein overload model in rats *in vivo*, we did demonstrate the protective role of PPARα agonists in an *in vitro* model of PTCs that were incubated with high doses of albumin and showed that PPARα agonists regulate megalin expression *in vivo* in mice. Interestingly, in mice, the hypolipidemic drugs ciprofibrate and WY 14643 exerted their effects at the level of megalin protein expression and did not increase megalin mRNA, in contrast to the upregulation of megalin mRNA that was induced in our cell culture model after a similar treatment. These somewhat contradictory results are not necessarily unexpected because differential activity of PPARα and its ligands on the expression of various genes has been reported, and it depends on the species of the analyzed gene [69], [70] and of the PPARα itself [70]. For example, in human, but not in rodent cells, the expression of the gene encoding for hydroxysteroid sulfotransferase (*sult2a1*) is induced in a PPARα dependent manner by ciprofibrate and WY 14643 [69]. In mice, the expression of the same enzyme is induced by WY 14643 in transgenic animals expressing human PPARα, but not in wild-type mice, indicating that only the human PPARα is able to activate the PPREs of the *sult2a1* gene. In our study, however, it is unlikely that the differential response of megalin mRNA expression observed in the mice is due to the PPARα species. Our experiments that tested the activation of the human megalin promoter by luciferase reporter assay and the EMSAs, which used human megalin probes, were performed with mouse PPARα. Moreover, the DNA-binding domains of human and mouse PPARα are completely homologous [70], suggesting that they should bind with similar affinities to the megalin PPREs. Another potential difference to consider is related to the numbers and sequences of PPREs in the megalin promoters of different species. Using Matinspector software (www.genomatix.de), we found that human, mouse and rat megalin promoters have different numbers and sequences of PPREs (data not shown). This difference could contribute to the absence of an induction of megalin mRNA expression by PPARα agonists in the mouse kidney *in vivo* and should be further explored in future studies.

Nevertheless, although megalin mRNA expression was not modified by the PPARα agonists ciprofibrate and WY 14643 *in vivo*, megalin protein levels were increased. Both agonists have been implicated in the regulation of the expression of several genes encoding inflammatory molecules, proteins related to lipid metabolism and transcription factors [71], [72]; several of these molecules could directly or indirectly affect megalin expression in the whole animal. It is also possible that turnover of the megalin protein is modified by PPARα agonists, leading to decreased degradation. For example, ciprofibrate, via a PPARα -dependent mechanism, has distinct roles in the expression of the HDL receptor, SR-BI; ciprofibrate increases SR-BI protein levels expression in murine and human macrophages [73] but reduces it in mouse liver [74]. On the other hand, it has been shown that fibrates activate different signaling pathways depending on ERK phosphorylation status, activation of PKC and activation of PI3K/AKT with inhibition of GSK3β [75], [76], [77], [78]. Accordingly, megalin expression is also regulated by signaling pathways depending on ERK and PI3K in PTCs [31], its proteolytic processing can be induced by PKC activation [79] and its cell surface expression is upregulated upon GSK3β inhibition [8]. Taken together, these data could indicate that multiple mechanisms may be operating to upregulate megalin expression in PPARα agonist-treated mice. Whatever the mechanism, similar to PPARγ agonists, PPARα agonists could be used as protective agents to reduce proteinuria and induce megalin expression.

PTC dysfunction with microalbuminuria is associated with early states of diabetes [26] and precedes glomerular damage [26]. These observations reinforce that maintenance of megalin expression, apical localization and trafficking in PTCs is critical for avoiding albuminuria and the subsequent renal damage, including inflammations and fibrosis. Furthermore, due to its role in signaling molecule degradation, megalin expression is required to negatively regulate the signaling of molecules involved in the early and late stages of renal disease, including angiotensin II [23] and leptin [22]; in addition to their other functions, these signaling molecules are involved in the regulation of renal inflammation and fibrosis and, for example, can induce the expression of TGFβ and Smad 2/3 activation [80], [81], [82], [83]. Other physiologically relevant roles of megalin include the tubular recovery of proteins such as vitamin D binding protein and liver fatty acid binding protein FABP1 [84]. Diabetic patients show an alteration in vitamin D metabolism, with lower levels of 1,25-OH vitamin D [85], due to an impairment in the activation of 25-OH vitamin D that occurs in the PTC after megalin-mediated endocytosis of the DBP-vitamin D complex. Moreover, treatment with PPARγ agonist pioglitazone reduced the urinary excretion of FABP1 in diabetic patients [86], which again reinforces the role of the PPAR system in the improvement of renal function through the activation of megalin expression.

In addition to the kidney, there are many other systems in which megalin function and expression are critical. During brain development, megalin expression is essential because it controls the sonic hedgehog and bone morphogenetic protein 4 signaling pathways by regulating their availability [5]. In the adult, megalin participates as an endocytic receptor in the gallbladder [3], blood-brain barrier [87] and nervous system, where it is associated with protection against neurodegeneration and with regenerative processes [16], [88]. Moreover, in all of these physiological systems, PPARs function as protective agents [89], [90], [91], [92], [93]. Thus, the significance of megalin/LRP2 expression in many organs is evident, and, therefore, it is important to understand the molecular and physiological regulation that controls expression of this the receptor. With our study, we added an important contribution to this field, showing that PPARα/γ are involved in the control of megalin expression. Our data indicate that, in addition to the previously proposed functions and target genes of PPARα/γ, the regulation of megalin expression and availability should also be considered an important role of the PPAR nuclear receptors.

## Materials and Methods

### Reagents

Dulbecco's Modified Eagle's Medium (DMEM), Minimum Essential Medium Alpha Modification (αMem), glutamine, PMSF, antipain, aprotinin, leupeptin, pepstatin, poly (dI-dC), BSA, DMSO and Triton X-100 were purchased from the Sigma Chemical Company (St. Louis, MO, USA). Fetal Bovine Serum (FBS) was from Hyclone (South Logan, UT, USA). Trypsin and Penicillin-Streptomycin were from Invitrogen/Gibco (Burlington, ON, USA). Lipofectamine 2000, random primers and RNAseOUT were from Invitrogen (Carlsbad, CA, USA). The Luciferase Reporter Assay and the TNT-coupled reticulocyte lysate system were from Promega (Madison, WI, USA). WY 14643 (PPARα agonist), GW 6471 (PPARα antagonist) and GW 9662 (PPARγ antagonist) were from Sigma. GW 610742 (PPARβ/δ agonist) was a gift from GlaxoSmithKline (Research Triangle Park, NC, USA), and rosiglitazone (PPARγ agonist) was from Cayman Chemical (Ann Arbor, MI, USA). BCA Protein Assay Reagent was from Pierce (Rockford, IL, USA). RNA-Solv reagent was from Omega Bio-Tek (Norcross, GA, USA). DNAseI, RevertAid M-MuLV Reverse Transcriptase, BamHI and XhoI were from Fermentas (Glen Burnie, MD, USA). Brilliant Sybr Green was from Stratagene (La Jolla, CA, USA). Oligonucleotide primers were obtained from Gene Link (Hawthorne, NY, USA). Telmisartan (SAMERTAN) was from BAGO (Santiago, Chile). Rosiglitazone (AVANDIA) and ciprofibrate (Estaprol) used for animal treatments were from GlaxoSmithKline (Mississauga, ON, Canada) and Sanofi Aventis (Amberes, France), respectively. Mouse monoclonal antibody to β-tubulin was purchased from Chemicon (Temecula, CA, USA). The polyclonal antiserum to recombinant human megalin cytoplasmic domain (anti-MegT) was previously described [7].The secondary antibodies for immunostaining and PAP complex were purchased from ICN Biomedicals, Inc. (Aurora, OH).

### Plasmids and constructs

(PPRE)_x3_TK-Luc, pCMX-mPPARα, pCMX-mPPARβ and pCMXm PPARγ were provided by Dr. R. M. Evans. pCMX·mRXRα was provided by Dr. M.Ananthanarayanan, and the -1500 pGL3-enhancer megalin promoter (−1500 meg prom) [94] was a gift from Dr. F. Lammert. TK-luc and pCMV-βvectors were from Clontech (Palo Alto, CA, USA). The pGL3 enhancer vector was from Promega. The -800 luciferase megalin promoter (−800 meg prom) was constructed as follows: −1500 meg prom was digested with BamHI and XhoI, and the resulting 930-bp fragment containing −830 to +100 (with +1 considered to be the transcriptional start site) of the megalin promoter was subcloned into a TK-Luc plasmid previously digested with BamHI and XhoI, which, therefore, lacked the TK promoter.

### Cell lines and treatments

LLC-PK1 and BN cells (both of which express endogenous megalin) were cultured essentially as described [3] and maintained at 37°C in 5% CO_2_. For testing PPAR agonists, BN and LLC-PK1 cells were cultured overnight in medium containing 0.5% FBS and then treated with agonists or vehicle (DMSO) for 24 h. For the experiments in which the PPAR antagonists were used, the cells were preincubated for 30 min before the addition of the respective agonists. BSA treatment in LLC-PK1 was performed in cells cultured overnight in medium depleted of serum. Then, various concentrations of BSA were added to the cells for 24 h. The treatments with PPAR agonists or the corresponding vehicle were performed 1 h before adding albumin at 20 mg/ml and were maintained for 24 h. At the end of the treatments, the cells were used for detection of protein by western blot or for RNA isolation. For western blots, the experiments were performed at least three times (n = 3), and for mRNA determination, each experimental condition was performed in triplicate with n = 3–4 independent experiments.

### Western blotting

Cells were lysed in PBS containing 1% Triton X-100, 2 mM PMSF, 1 mM pepstatin, antipain, 1 µM leupeptin and 0.3 µM aprotinin. Kidney extracts were obtained as indicated below. Equal amounts of protein from each extract were subjected to SDS-PAGE under reducing conditions. The immunodetection of megalin, tubulin or actin was determined as described [3], [7]. Densitometric analysis was performed using the Gel Doc 2000 Gel Documentation System and Quantity One version 4 software (Bio-Rad, CA, USA). The signal generated by β-tubulin immunolabeling was used for normalization.

### Megalin mRNA quantification by real-time PCR (qPCR)

Total RNA was isolated using RNA-Solv and following the manufacturer's instructions. Total RNA (1 µg) was first digested by DNAse I and then subjected to reverse transcription in a 20 µl reaction mixture using random primers and RevertAid™ M-MuLV Reverse Transcriptase in the presence of RNAseOUT. PCR reactions were performed using a 7500 Real-Time PCR System (Applied Biosystems, Carlsbad, CA, USA). TaqMan gene expression assay primers were used for mouse megalin, Acox and GADPH (Applied Biosystems). For the other mRNAs, Brilliant SybrGreen I (Stratagene) was used (see Table S1 for primers). All primers were used at a final concentration of 300 nM. Analyses of results were performed with the 7500 System Software.

### Promoter and reporter assays

BN and LLC-PK1 cells were cotransfected with 300 ng of −800 meg-Luc and 300 ng each of either PPARα or PPARγ cDNA. Control wells were supplemented with the same amount of vector DNA such that the total amount of DNA added to each well remained constant. All cells were also transfected with a β-galactosidase vector (pCMVβ) as an internal control for transfection efficiency. On the second day, cells were treated with the appropriate PPAR agonist for 24 h in their respective medium with 0.5% FBS. Luciferase activity was assayed in a luminometer using the Luciferase Reporter Assay System (Promega) according to the manufacturer's instructions. Luciferase activity was normalized for transfection efficiency using the expression of β-galactosidase. Results are expressed as the fold increase of activity over the control vector.

### Electrophorectic mobility shift assays

Using the TNT-coupled reticulocyte lysate system (Promega), cDNAs encoding mouse RXRα, PPARα, PPARβ and PPARγ were transcribed and translated according to the manufacturer's instructions. Aliquots of the transcribed/translated extracts were used for EMSAs. Reactions (2 µl) containing PPARα, PPARβ or PPARγ alone or together with RXRα were added to 9 µl total volume of binding buffer (10 mM Tris HCl, 50 mM NaCl, 0.5 mM dithiothreitol, 0.5 mM EDTA, 1 mM MgCl_2_, 4% glycerol, 0.5 mg/ml poly (dI-dC) at 25°C for 15 min followed by another 20 min incubation with 10 µCi of [γ-^32^P]ATP-labeled oligonucleotide containing the putative human PPRE binding sites. For competition assays, unlabeled wild-type or mutant oligonucleotides were added to the reaction 15 min before the addition of the probe. For probe details, see Table S2. The DNA-protein binding complex was run on a 4% non-denatured polyacrylamide gel in 0.25× TBE at 220 V for 1.5 h. Gels were autoradiographed using Kodak Biomax film.

### Animals and experimental procedures

Animal protocols were carried out with approval from the Review Board for animal studies at the Facultad de Ciencias Biológicas and the Ethical Committee of the Facultad de Medicina, Pontificia Universidad Católica de Chile (Approval Certificate CE #020106) and according to the Guide for the Care and Use of Laboratory Animals of CONICYT. Animals were maintained at constant room temperature with a 12-h light/dark cycle.

#### Ciprofibrate, WY 14643, rosiglitazone and Telmisartan treatment in mice (see Table S3 for more details)

The kidneys were harvested and washed in PBS 24 h after finishing the treatment protocols. One kidney was processed for immunohistochemistry (as described below), and the other was used for western blot and qPCR analyses. For western blots, one kidney half was homogenized on ice in homogenization buffer (PBS, 0.5% (v/v) Triton X-100, 2 mM PMSF, 1 mM pepstatin, antipain, 1 µM leupeptin and 0.3 µM aprotinin) using a dounce homogenizer; nuclear debris was removed by centrifugation at 3,000× *g* for 10 min at 4°C, and the supernatants were saved. For qPCR assays, total RNA was isolated from the remaining kidney half using RNA-Solv reagent (Omega-BioTek) according to the manufacturer's instructions.

#### Induction of tubulointerstitial damage and proteinuria in Sprague-Dawley rats (see Table S4 for more details)

After treatments were finished, kidneys were removed for morphologic and qPCR analyses from the rats while they were under anesthesia (ketamine/xylazine). To determine the renal function levels, animals were subjected to urine and blood collections. Urine collections were performed in individual metabolic cages for 16 h, during which time food was withheld to avoid fecal contamination, but water was offered ad libitum. Functional damage was assessed by the determination of serum creatinine levels and creatinine clearance. Urine and plasma creatinine were assayed in a Beckman Creatinine analyzer (Beckman Coulter, Fullerton, CA). Creatinine clearance over 16 h was calculated according to the standard formula *C*  =  *U*x*V*/P, where *C* is creatinine clearance, *U* is creatinine urinary concentration, *V* is the urine flow rate per minute and *P* is creatinine plasmatic concentration [95].

### Tissue processing and immunohistochemical analysis

Renal tissue samples from the different groups (3 mm thick) were fixed by immersion in Bouin's solution for 24 h at room temperature. The tissue was then dehydrated, embedded in Paraplast Plus (Monoject Scientific, St. Louis, MO), serially sectioned at 5-um thickness with a rotatory microtome, mounted on glass slides and used for immunostaining. Immunostaining was performed using an indirect immunoperoxidase technique [96], [97] to localize megalin in rat or mouse kidneys. Briefly, tissue sections were dewaxed, rehydrated, rinsed in 0.05 M Tris-phosphate-saline (TPS) buffer pH 7.6 and incubated with the primary antiserum raised against megalin 1:120,000 overnight at 22°C. The secondary antibody and the corresponding antiperoxidase (PAP) complex were applied for 30 min each at 22°C. The immunoperoxidase reaction was visualized by incubating sections in 0.1% (wt/vol) 3,3′-diaminobenzidine and 0.03% hydrogen peroxide. The antisera and PAP complex were diluted in TPS buffer containing 0.25% Triton X-100 and 0.7% (wt/vol) λ-carrageenan. Controls for the immunostaining procedure were prepared by omission of the first antibody or by the replacement with the preimmune serum. The sections were rinsed with TPS buffer between incubations, counterstained with hematoxylin, dehydrated and cleared with xylene and then coverslipped. The tissue samples from all of the groups were coded and studied independently by 2 observers (C.P.V and C.C.) in a blinded fashion; sections selected at random were used for each animal, and 3–4 fields per section were studied. Images were acquired using a Nikon Eclipse E600 microscope and Nikon DXM1200 digital camera. The megalin-stained area in each image was determined utilizing computer-assisted image analysis software, Simple PCI (Hamamatsu Co, PA USA). The values corresponding to total immunostained (brown) cells were averaged and expressed as the mean absolute values and expressed as squared microns as described [98]. To evaluate the tubulointerstitial damage in rats, focus was placed on tubular shape, the presence of protein casts within the lumens of tubules, cell infiltration in the interstitium and immunostaining as previously described [98].

### Statistical analysis

Data are expressed as means ± SD of at least three independent experiments. Comparisons of three or more groups were performed using ANOVA followed by the Bonferroni test. An unpaired Student's *t*-test was used when two groups were compared.

## Supporting Information

Figure S1 **Detection of PPARα, PPARβ/δ and PPARγ in BN cells by RT-PCR.** Expression of the PPAR (α, β and γ) nuclear receptors in the rat yolk sac cell line BN was analyzed by RT-PCR using different numbers of cycles. The presence of the PPAR mRNAs in the cell extracts is evident. These expression levels were compared to those observed in the rat liver (right panels). In the liver samples, the bands shown correspond to 26 cycles for PPARα, 29 cycles for PPARβ and 25 cycles for PPARγ.
(TIF)

Figure S2 **Mice receiving ciprofibrate and WY 14643 respond to the activation of PPARα but only augment the expression of megalin at the protein level.** BALB/c mice (n = 3-5/per group) received ciprofibrate (200 mg/kg/day) or vehicle for 1 week or W Y14643 (50 mg/kg/day) or vehicle for 10 days. (**a**) The effectiveness of the PPARα agonists was confirmed by quantification of the mRNA levels of the PPARα target gene Acox by qPCR. (**b**) WY 14643 did not affect the expression of megalin mRNA. (**c**) Kidney extracts from the animals treated with WY 14634 were used to determine megalin protein levels by western blot, with β-tubulin used as loading control. (**d**) The bands in the blots were quantified by densitometry, and the results were plotted as the ratio of megalin/β-tubulin for each condition. Results are expressed as the mean ± standard deviation (SD). Statistically significant differences are indicated by *P < 0.05 vs. control, **P < 0.01 vs. control.
(TIF)

Figure S3 **The expression of megalin is induced by telmisartan in mouse and rat kidney.** BALB/c mice (n = 4-6/group) and Sprague-Dawley rats (n = 4/group) were treated for 7 or 4 days, respectively, with telmisartan (3 mg/kg/day) or vehicle. (**a**) Kidney extracts were used to determine megalin protein levels in mice, with β-tubulin used as loading control. (**b**) The bands in the blots were quantified by densitometry, and the results were plotted as the ratio of megalin/β-tubulin for each condition. (**c,d**) Immunohistochemical detection of megalin protein in rat kidney sections (cortex) was performed and quantified. The plots show the megalin staining area (µm^2^), which was significantly increased in the telmisartan group compared to the control group. Results are expressed as means ± standard deviation (SD). Statistically significant differences are indicated as *P<0.05.
(TIF)

Table S1 **Primers for qPCR**
(DOC)

Table S2 **Probes for EMSA**
(DOCX)

Table S3 **PPARα and PPARγ agonist treatment in mice**
(DOCX)

Table S4 **Protocol for inducing tubulointerstitial damage and proteinuria in rats; the role of PPARγ agonists in megalin expression**
(DOCX)
