# Supplementary figures and images for: Megalin/LRP2 Expression Is Induced by Peroxisome Proliferator-Activated Receptor -Alpha and -Gamma: Implications for PPARs' Roles in Renal Function
Source: PLoS One. 2011 Feb 2;6(2):e16794. doi: 10.1371/journal.pone.0016794 (PMC3032793; doi:10.1371/journal.pone.0016794)

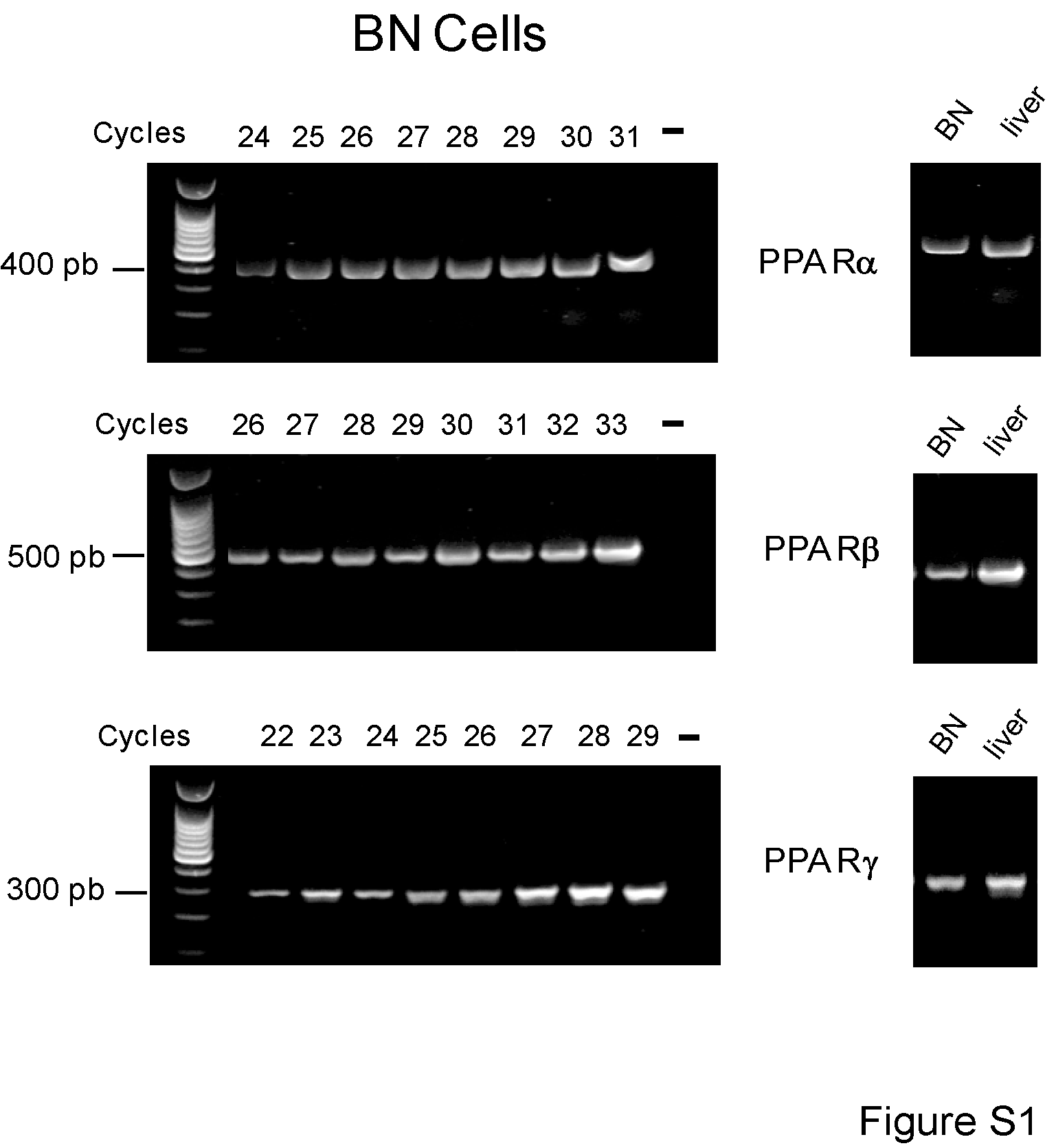

Supplement: Figure S1 — Detection of PPARα, PPARβ/δ and PPARγ in BN cells by RT-PCR. Expression of the PPAR (α, β and γ) nuclear receptors in the rat yolk sac cell line BN was analyzed by RT-PCR using different numbers of cycles. The presence of the PPAR mRNAs in the cell extracts is evident. These expression levels were compared to those observed in the rat liver (right panels). In the liver samples, the bands shown correspond to 26 cycles for PPARα, 29 cycles for PPARβ and 25 cycles for PPARγ. (TIF) [file pone.0016794.s001.tif]

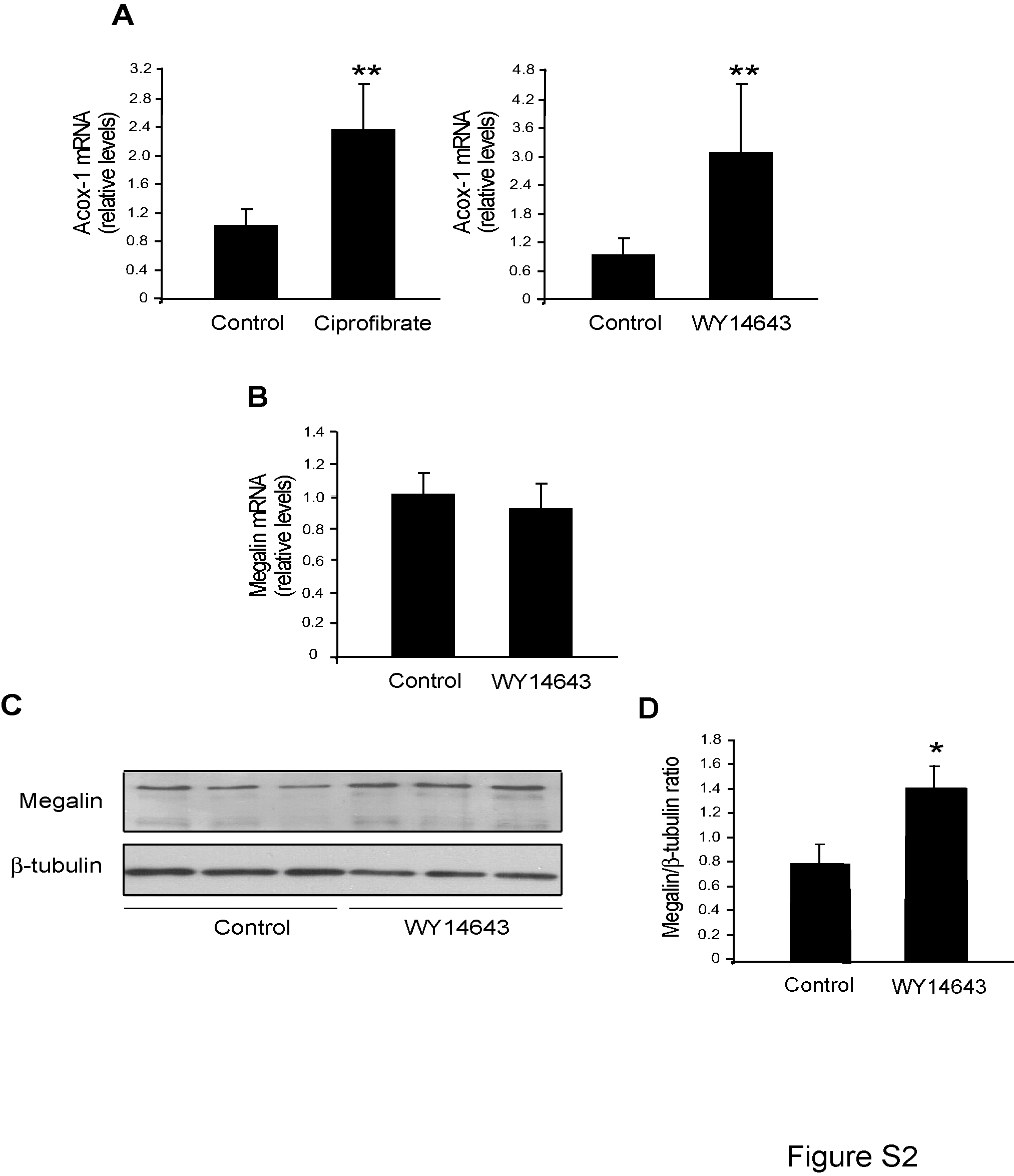

Supplement: Figure S2 — Mice receiving ciprofibrate and WY 14643 respond to the activation of PPARα but only augment the expression of megalin at the protein level. BALB/c mice (n = 3-5/per group) received ciprofibrate (200 mg/kg/day) or vehicle for 1 week or W Y14643 (50 mg/kg/day) or vehicle for 10 days. (a) The effectiveness of the PPARα agonists was confirmed by quantification of the mRNA levels of the PPARα target gene Acox by qPCR. (b) WY 14643 did not affect the expression of megalin mRNA. (c) Kidney extracts from the animals treated with WY 14634 were used to determine megalin protein levels by western blot, with β-tubulin used as loading control. (d) The bands in the blots were quantified by densitometry, and the results were plotted as the ratio of megalin/β-tubulin for each condition. Results are expressed as the mean ± standard deviation (SD). Statistically significant differences are indicated by *P < 0.05 vs. control, **P < 0.01 vs. control. (TIF) [file pone.0016794.s002.tif]

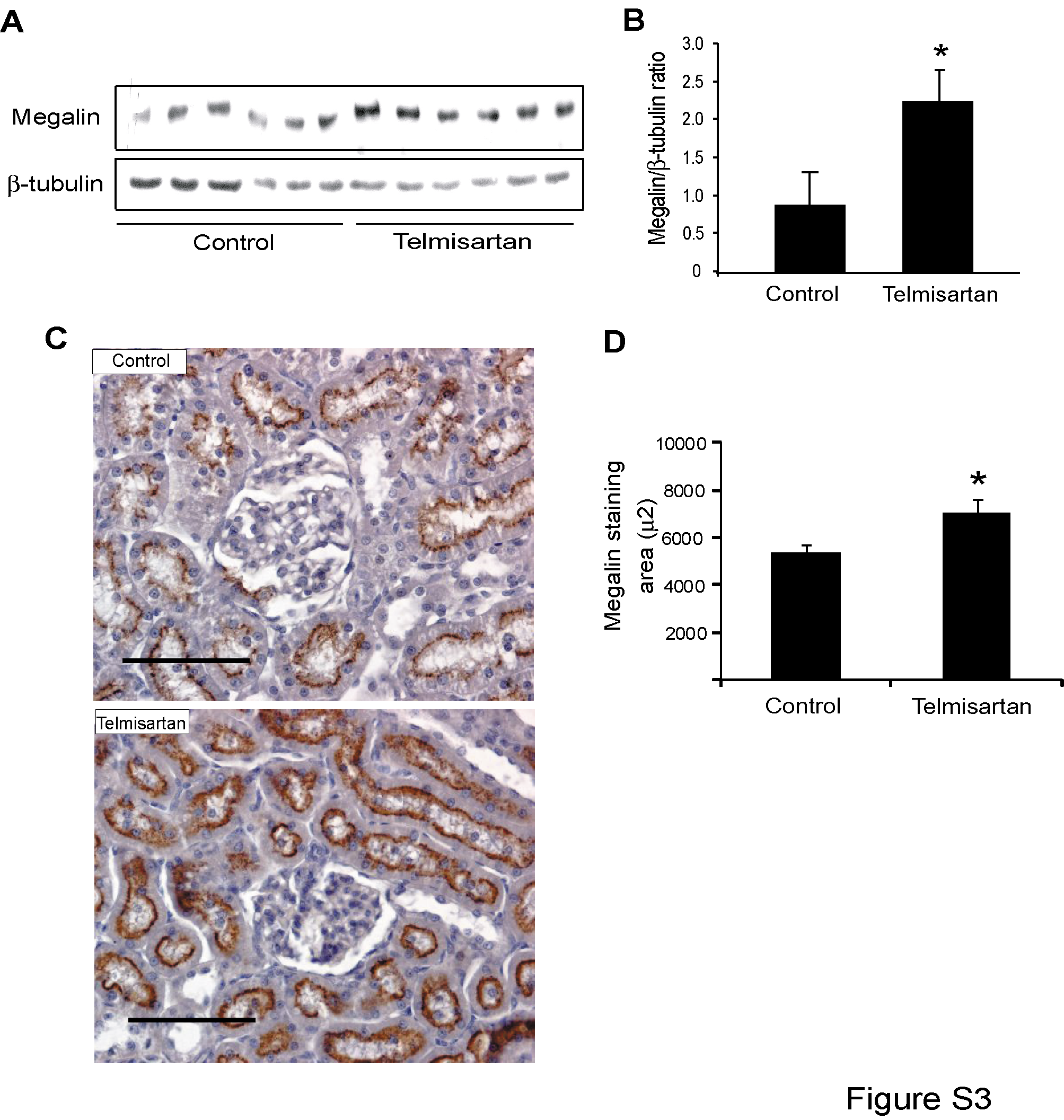

Supplement: Figure S3 — The expression of megalin is induced by telmisartan in mouse and rat kidney. BALB/c mice (n = 4-6/group) and Sprague-Dawley rats (n = 4/group) were treated for 7 or 4 days, respectively, with telmisartan (3 mg/kg/day) or vehicle. (a) Kidney extracts were used to determine megalin protein levels in mice, with β-tubulin used as loading control. (b) The bands in the blots were quantified by densitometry, and the results were plotted as the ratio of megalin/β-tubulin for each condition. (c,d) Immunohistochemical detection of megalin protein in rat kidney sections (cortex) was performed and quantified. The plots show the megalin staining area (µm2), which was significantly increased in the telmisartan group compared to the control group. Results are expressed as means ± standard deviation (SD). Statistically significant differences are indicated as *P<0.05. (TIF) [file pone.0016794.s003.tif]
