# Supplementary material for: Megalin/LRP2 Expression Is Induced by Peroxisome Proliferator-Activated Receptor -Alpha and -Gamma: Implications for PPARs' Roles in Renal Function
Source: PLoS One. 2011 Feb 2;6(2):e16794. doi: 10.1371/journal.pone.0016794 (PMC3032793; doi:10.1371/journal.pone.0016794)
Supplement: Table S1 — Primers for qPCR (DOC) [file pone.0016794.s004.doc]

| **Gene** | **Direction** | **Primer** |
| --- | --- | --- |
| Rat Megalin | Sense | GATGCTGTTGCTGGCGATCG |
|  | Antisense | CATTGTCACAGCGAAAATTCCCAC |
| Rat β-actin | Sense | GCATGTGCAAGGCCGGCT |
|  | Antisense | CACCATCACACCCTGGTGCCTA |
| Pig Megalin |  | CTGCTCTTGTAGACCTGGGTTC |
|  | Antisense | TCGGCACAGCTACACTCATAAC |
| pig Acox | Sense | CTCGCAGACCCAGATGAAAT |
|  | Antisense | TCCAAGCCTCGAAGATGAGT |
| Pig Caveolin1 | Sense | ACAAGCCCAACAACAAGGCCA |
|  | Antisense | ttcgtcacagtgaaggtggtg |
| Pig Actin | Sense | CCAGATCATGTTCGAGACCTTC |
|  | Antisense | TCTTCATGAGGTAGTCGGTCAG |

**Table S1.** Primers for qPCR
