# Supplementary material for: Megalin/LRP2 Expression Is Induced by Peroxisome Proliferator-Activated Receptor -Alpha and -Gamma: Implications for PPARs' Roles in Renal Function
Source: PLoS One. 2011 Feb 2;6(2):e16794. doi: 10.1371/journal.pone.0016794 (PMC3032793; doi:10.1371/journal.pone.0016794)
Supplement: Table S2 — Probes for EMSA (DOCX) [file pone.0016794.s005.docx]

**Table S2.** Probes for EMSA

| **Sites** | **Probes** |
| --- | --- |
| -700 wt | CTAGAGAGATCAGGCCAGGAATGAAGGTCAC AGATCTCCATCCAG |
| -2150 wt | CTGGCTGCTACAGTGAAAGGGCACACACA |
| -2800 wt | GCTTGGCTTCTGGGGCAATGGTGGTGCTGAA |
| -700mut | CTAGAGAGATCAGGC***GG***GGAATGAAGGT***GG***C AGATCTCCATCCAG |
| -2100 mut | CTGGCTGCTACAGTGA***CC***GGGCACACACA |
| -2800 mut | GCTTGGCTTCT***CC***GGCAAT***CC***TGGTGCTGAA |
