# Supplementary material for: Megalin/LRP2 Expression Is Induced by Peroxisome Proliferator-Activated Receptor -Alpha and -Gamma: Implications for PPARs' Roles in Renal Function
Source: PLoS One. 2011 Feb 2;6(2):e16794. doi: 10.1371/journal.pone.0016794 (PMC3032793; doi:10.1371/journal.pone.0016794)
Supplement: Table S3 — PPARα and PPARγ agonist treatment in mice (DOCX) [file pone.0016794.s006.docx]

**Table S3**. PPAR α and γ agonist treatment in mice

| **Group** | **N** | **Dose (mg/kg/day)** | **Vehicle** | **Days of treatment** |
| --- | --- | --- | --- | --- |
| Control | 5 | - | Corn oil | 7 |
| Ciprofibrate | 5 | 200 | Corn oil | 7 |
| Control | 3 | - | 20% Tween80/ 80% PEG400 | 10 |
| WY14643 | 3 | 50 | 20% Tween80/ 80% PEG400 | 10 |
| Control | 4 | - | 20% Tween80/ 80% PEG400 | 10 |
| Rosiglitazone | 4 | 20 | 20% Tween80/ 80% PEG400 | 10 |
| Control | 6 | - | 20% Tween80/ 80% PEG400 | 7 |
| Telmisartan | 6 | 3 | 20% Tween80/ 80% PEG400 | 7 |

At the beginning of the treatments all mice were 8 weeks of age. All the drugs were given orally by gavage.
