# Supplementary material for: Megalin/LRP2 Expression Is Induced by Peroxisome Proliferator-Activated Receptor -Alpha and -Gamma: Implications for PPARs' Roles in Renal Function
Source: PLoS One. 2011 Feb 2;6(2):e16794. doi: 10.1371/journal.pone.0016794 (PMC3032793; doi:10.1371/journal.pone.0016794)
Supplement: Table S4 — Protocol for inducing tubulointerstitial damage and proteinuria in rats; the role of PPARγ agonists in megalin expression (DOCX) [file pone.0016794.s007.docx]

**Table S4**. **Protocol for inducing tubulointerstitial damage and proteinuria in rats;**

**the role of PPARγ agonists in megalin expression**

| **Group** | **N** | **Dose** (mg/kg/day)** | **Days of treatment** |
| --- | --- | --- | --- |
| Control | 3 | # | 4 |
| Telmisartan | 3 | 3 | 4 |
| Rosiglitazone | 3 | 3 | 4 |
| Control & | 4 | # | 11 |
| BSA * | 4 | # | 11 |
| Telmisartan + BSA * | 4 | 3 | 11 |
| Rosiglitazone + BSA* | 4 | 3 | 11 |

All the rats were 7 weeks of age at the beginning of the experiment. At day 4, the three first groups were sacrificed and their kidneys removed for morphologic analysis and qPCR. On day 4 and 11, animals that received gavage and I.P. injections were subjected to urine and blood collections to measure proteinuria, serum creatinine levels and creatinine clearence. On day 12 kidneys were removed for morphologic analysis and qPCR. ** Drug dissolved in vehicle (0.5% methylcellulose/0.025% Tween20 dissolved in 0.9% sterile saline) and given by gavage; # Vehicle (0.5% methylcellulose/0.025% Tween20 dissolved in 0.9% sterile saline) given by gavage; & 0.9% sterile saline I.P. from day 5 to 11; * BSA 2g/day in 0.9% sterile saline; I.P. from day 5 to 11.
